# A User's Guide to the Encyclopedia of DNA Elements (ENCODE)

**DOI:** 10.1371/journal.pbio.1001046

**Published:** 2011-04-19

**Authors:** The ENCODE Project Consortium

**Affiliations:** Adolf Butenandt Institute, Germany

## Abstract

The mission of the Encyclopedia of DNA Elements (ENCODE) Project is to enable the scientific and medical communities to interpret the human genome sequence and apply it to understand human biology and improve health. The ENCODE Consortium is integrating multiple technologies and approaches in a collective effort to discover and define the functional elements encoded in the human genome, including genes, transcripts, and transcriptional regulatory regions, together with their attendant chromatin states and DNA methylation patterns. In the process, standards to ensure high-quality data have been implemented, and novel algorithms have been developed to facilitate analysis. Data and derived results are made available through a freely accessible database. Here we provide an overview of the project and the resources it is generating and illustrate the application of ENCODE data to interpret the human genome.

## Author Summary

The Encyclopedia of DNA Elements (ENCODE) Project was created to enable the scientific and medical communities to interpret the human genome sequence and to use it to understand human biology and improve health. The ENCODE Consortium, a large group of scientists from around the world, uses a variety of experimental methods to identify and describe the regions of the 3 billion base-pair human genome that are important for function. Using experimental, computational, and statistical analyses, we aimed to discover and describe genes, transcripts, and transcriptional regulatory regions, as well as DNA binding proteins that interact with regulatory regions in the genome, including transcription factors, different versions of histones and other markers, and DNA methylation patterns that define states of the genome in various cell types. The ENCODE Project has developed standards for each experiment type to ensure high-quality, reproducible data and novel algorithms to facilitate analysis. All data and derived results are made available through a freely accessible database. This article provides an overview of the complete project and the resources it is generating, as well as examples to illustrate the application of ENCODE data as a user's guide to facilitate the interpretation of the human genome.

## I. Introduction and Project Overview

Interpreting the human genome sequence is one of the leading challenges of 21^st^ century biology [1]. In 2003, the National Human Genome Research Institute (NHGRI) embarked on an ambitious project—the Encyclopedia of DNA Elements (ENCODE)—aiming to delineate all of the functional elements encoded in the human genome sequence [2]. To further this goal, NHGRI organized the ENCODE Consortium, an international group of investigators with diverse backgrounds and expertise in production and analysis of high-throughput functional genomic data. In a pilot project phase spanning 2003–2007, the Consortium applied and compared a variety of experimental and computational methods to annotate functional elements in a defined 1% of the human genome [3]. Two additional goals of the pilot ENCODE Project were to develop and advance technologies for annotating the human genome, with the combined aims of achieving higher accuracy, completeness, and cost-effective throughput and establishing a paradigm for sharing functional genomics data. In 2007, the ENCODE Project was expanded to study the entire human genome, capitalizing on experimental and computational technology developments during the pilot project period. Here we describe this expanded project, which we refer to throughout as the ENCODE Project, or ENCODE.

The major goal of ENCODE is to provide the scientific community with high-quality, comprehensive annotations of candidate functional elements in the human genome. For the purposes of this article, the term “functional element” is used to denote a discrete region of the genome that encodes a defined product (e.g., protein) or a reproducible biochemical signature, such as transcription or a specific chromatin structure. It is now widely appreciated that such signatures, either alone or in combinations, mark genomic sequences with important functions, including exons, sites of RNA processing, and transcriptional regulatory elements such as promoters, enhancers, silencers, and insulators. However, it is also important to recognize that while certain biochemical signatures may be associated with specific functions, our present state of knowledge may not yet permit definitive declaration of the ultimate biological role(s), function(s), or mechanism(s) of action of any given genomic element.

At present, the proportion of the human genome that encodes functional elements is unknown. Estimates based on comparative genomic analyses suggest that 3%–8% of the base pairs in the human genome are under purifying (or negative) selection [4]–[7]. However, this likely underestimates the prevalence of functional features, as current comparative methods may not account for lineage-specific evolutionary innovations, functional elements that are very small or fragmented [8], elements that are rapidly evolving or subject to nearly neutral evolutionary processes, or elements that lie in repetitive regions of the genome.

**Figure 1:**
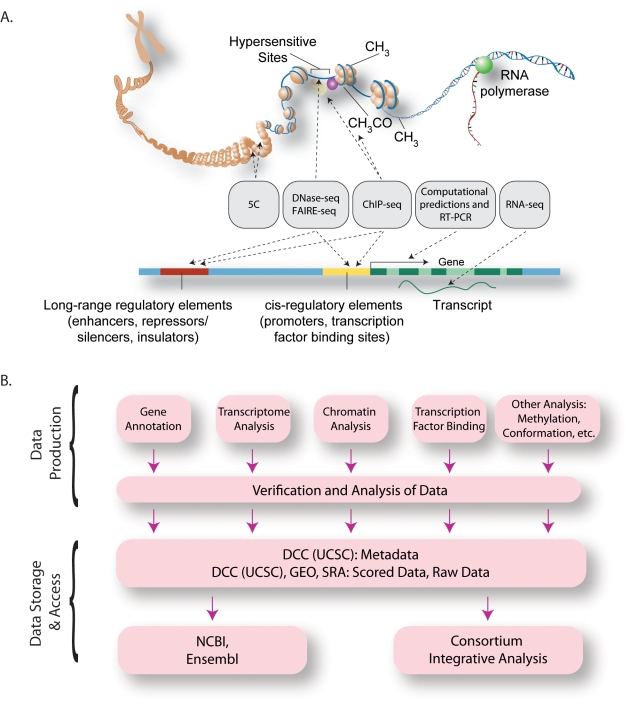
The Organization of the ENCODE Consortium. (A) Schematic representation of the major methods that are being used to detect functional elements (gray boxes), represented on an idealized model of mammalian chromatin and a mammalian gene. (B) The overall data flow from the production groups after reproducibility assessment to the Data Coordinating Center (UCSC) for public access and to other public databases. Data analysis is performed by production groups for quality control and research, as well as at a cross-Consortium level for data integration.

**Figure 2:**
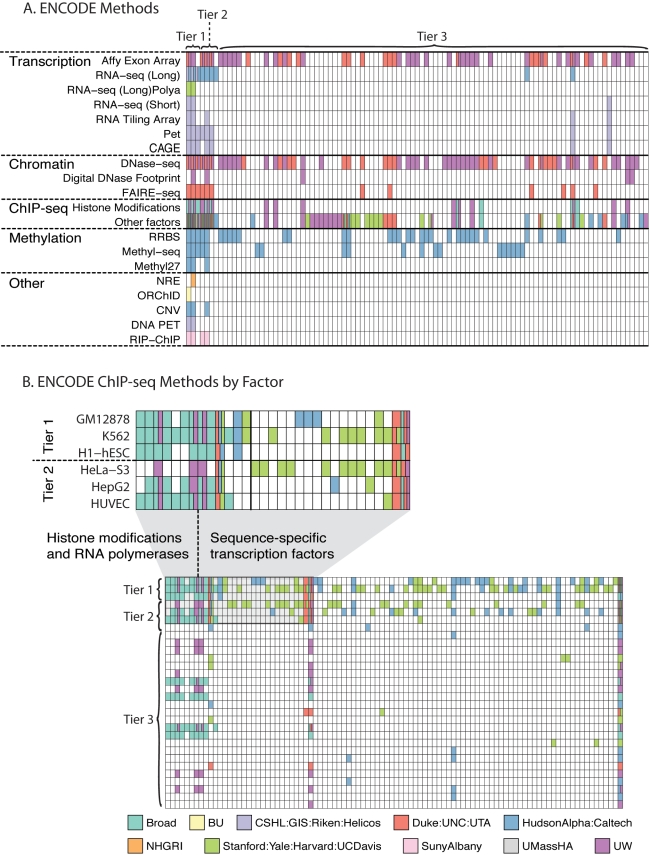
Data available from the ENCODE Consortium. (A) A data matrix representing all ENCODE data types. Each row is a method and each column is a cell line on which the method could be applied to generate data. Colored cells indicate that data have been generated for that method on that cell line. The different colors represent data generated from different groups in the Consortium as indicated by the key at the bottom of the figure. In some cases, more than one group has generated equivalent data; these cases are indicated by subdivision of the cell to accommodate multiple colors. (B) Data generated by ChIP-seq are split into a second matrix where the cells now represent cell types (rows) split by the factor or histone modification to which the antibody is raised (columns). The colors again represent the groups as indicated by the key. The upper left corner of this matrix has been expanded immediately above the panel to better illustrate the data. All data were collected from the ENCODE public download repository at http://hgdownload.cse.ucsc.edu/goldenPath/hg18/encodeDCC on September 1, 2010.

The current phase of the ENCODE Project has focused on completing two major classes of annotations: genes (both protein-coding and non-coding) and their RNA transcripts, and transcriptional regulatory regions. To accomplish these goals, seven ENCODE Data Production Centers encompassing 27 institutions have been organized to focus on generating multiple complementary types of genome-wide data (Figure 1 and Figure S1). These data include identification and quantification of RNA species in whole cells and in sub-cellular compartments, mapping of protein-coding regions, delineation of chromatin and DNA accessibility and structure with nucleases and chemical probes, mapping of histone modifications and transcription factor (TF) binding sites by chromatin immunoprecipitation (ChIP), and measurement of DNA methylation (Figure 2 and Table 1). In parallel with the major production efforts, several smaller-scale efforts are examining long-range chromatin interactions, localizing binding proteins on RNA, identifying transcriptional silencer elements, and understanding detailed promoter sequence architecture in a subset of the genome (Figure 1 and Table 1).

**Table 1:** Experimental assays used by the ENCODE Consortium.

Gene/Transcript Analysis		
Region/Feature	Method	Group
Gene annotation	GENCODE	Wellcome Trust
PolyA+ coding regions	RNA-seq; tiling DNA microarrays; PET	CSHL; Stanford/Yale//Harvard; Caltech
Total RNA coding regions	RNA-seq; tiling DNA microarrays; PET	CSHL
Coding regions in subcellular RNA fractions (e.g. nuclear, cytoplasmic)	PET	CSHL
Small RNAs	short RNA-seq	CSHL
Transcription initiation (5′-end) and termination (3-end′) sites	CAGE; diTAGs	RIKEN, GIS
Full-length RNAs	RACE	University of Geneva; University of Lausanne
Protein-bound RNA coding regions	RIP; CLIP	SUNY-Albany; CSHL

Transcription Factors/Chromatin		
Elements/Regions	Method(s)	Group(s)
Transcription Factor Binding Sites (TFBS)	ChIP-seq	Stanford/Yale/UC-Davis/Harvard; HudsonAlpha/Caltech; Duke/UT-Austin; UW; U. Chicago/Stanford
Chromatin structure (accessibility, etc.)	DNaseI hypersensitivity; FAIRE	UW; Duke; UNC
Chromatin modifications (H3K27ac, H3K27me3, H3K36me3, etc.)	ChIP-seq	Broad; UW
DNaseI footprints	Digital genomic footprinting	UW

Other Elements/Features		
Feature	Method(s)	Group(s)
DNA methylation	RRBS; Illumina Methyl27; Methyl-seq	HudsonAlpha
Chromatin interactions	5C; CHIA-PET	UMass; UW; GIS
Genotyping	Illumina 1M Duo	HudsonAlpha

ENCODE has placed emphasis on data quality, including ongoing development and application of standards for data reproducibility and the collection of associated experimental information (i.e., metadata). Adoption of state-of-the-art, massively parallel DNA sequence analysis technologies has greatly facilitated standardized data processing, comparison, and integration [9],[10]. Primary and processed data, as well as relevant experimental methods and parameters, are collected by a central Data Coordination Center (DCC) for curation, quality review, visualization, and dissemination (Figure 1). The Consortium releases data rapidly to the public through a web-accessible database (http://genome.ucsc.edu/ENCODE/) [11] and provides a visualization framework and analytical tools to facilitate use of the data [12], which are organized into a web portal (http://encodeproject.org).

To facilitate comparison and integration of data, ENCODE data production efforts have prioritized selected sets of cell types (Table 2). The highest priority set (designated “Tier 1”) includes two widely studied immortalized cell lines—K562 erythroleukemia cells [13]; an EBV-immortalized B-lymphoblastoid line (GM12878, also being studied by the 1,000 Genomes Project; http://1000genomes.org) and the H1 human embryonic stem cell line [14]. A secondary priority set (Tier 2) includes HeLa-S3 cervical carcinoma cells [15], HepG2 hepatoblastoma cells [16], and primary (non-transformed) human umbilical vein endothelial cells (HUVEC; [17]), which have limited proliferation potential in culture. To capture a broader spectrum of human biological diversity, a third set (Tier 3) currently comprises more than 100 cell types that are being analyzed in selected assays (Table 2). Standardized growth conditions for all ENCODE cell types have been established and are available through the ENCODE web portal (http://encodeproject.org, “cell types” link).

**Table 2:** ENCODE cell types.

Cell Type	Tier	Description	Source
GM12878	1	B-Lymphoblastoid cell line	Coriell GM12878
K562	1	Chronic Myelogenous/Erythroleukemia cell line	ATCC CCL-243
H1-hESC	1	Human Embryonic Stem Cells, line H1	Cellular Dynamics International
HepG2	2	Hepatoblastoma cell line	ATCC HB-8065
HeLa-S3	2	Cervical carcinoma cell line	ATCC CCL-2.2
HUVEC	2	Human Umbilical Vein Endothelial Cells	Lonza CC-2517
Various (Tier 3)	3	Various cell lines, cultured primary cells, and primary tissues	Various

This report is intended to provide a guide to the data and resources generated by the ENCODE Project to date on Tier 1–3 cell types. We summarize the current state of ENCODE by describing the experimental and computational approaches used to generate and analyze data. In addition, we outline how to access datasets and provide examples of their use.

## II. ENCODE Project Data

The following sections describe the different types of data being produced by the ENCODE Project (Table 1).

### Genes and Transcripts

#### Gene annotation

A major goal of ENCODE is to annotate all protein-coding genes, pseudogenes, and non-coding transcribed loci in the human genome and to catalog the products of transcription including splice isoforms. Although the human genome contains ∼20,000 protein-coding genes [18], accurate identification of all protein-coding transcripts has not been straightforward. Annotation of pseudogenes and noncoding transcripts also remains a considerable challenge. While automatic gene annotation algorithms have been developed, manual curation remains the approach that delivers the highest level of accuracy, completeness, and stability [19]. The ENCODE Consortium has therefore primarily relied on manual curation with moderate implementation of automated algorithms to produce gene and transcript models that can be verified by traditional experimental and analytical methods. This annotation process involves consolidation of all evidence of transcripts (cDNA, EST sequences) and proteins from public databases, followed by building gene structures based on supporting experimental data [20]. More than 50% of annotated transcripts have no predicted coding potential and are classified by ENCODE into different transcript categories. A classification that summarizes the certainty and types of the annotated structures is provided for each transcript (see http://www.gencodegenes.org/biotypes.html for details). The annotation also includes extensive experimental validation by RT-PCR for novel transcribed loci (i.e., those not previously observed and deposited into public curated databases such as RefSeq). Pseudogenes are identified primarily by a combination of similarity to other protein-coding genes and an obvious functional disablement such as an in-frame stop codon. Because it is difficult to validate pseudogenes experimentally, three independent annotation methods from Yale (“pseudopipe”) [21], UCSC (“retrofinder”; http://users.soe.ucsc.edu/~markd/gene-sets-new/pseudoGenes/RetroFinder.html, and references therein), and the Sanger Center [20] are combined to produce a consensus pseudogene set. Ultimately, each gene or transcript model is assigned one of three confidence levels. Level 1 includes genes validated by RT-PCR and sequencing, plus consensus pseudogenes. Level 2 includes manually annotated coding and long non-coding loci that have transcriptional evidence in EMBL/GenBank. Level 3 includes Ensembl gene predictions in regions not yet manually annotated or for which there is new transcriptional evidence.

The result of ENCODE gene annotation (termed “GENCODE”) is a comprehensive catalog of transcripts and gene models. ENCODE gene and transcript annotations are updated bimonthly and are available through the UCSC ENCODE browser, distributed annotation servers (DAS; see http://genome.ucsc.edu/cgi-bin/das/hg18/features?segment=21:33031597,33041570type=wgEncodeGencodeManualV3), and the Ensembl Browser [22].

#### RNA transcripts

ENCODE aims to produce a comprehensive genome-wide catalog of transcribed loci that characterizes the size, polyadenylation status, and subcellular compartmentalization of all transcripts (Table 1).

ENCODE has generated transcript data with high-density (5 bp) tiling DNA microarrays [23] and massively parallel DNA sequencing methods [9],[10],[24], with the latter predominating in ongoing efforts. Both polyA+ and polyA− RNAs are being analyzed. Because subcellular compartmentalization of RNAs is important in RNA processing and function, such as nuclear retention of unspliced coding transcripts [25] or snoRNA activity in the nucleolus [26], ENCODE is analyzing not only total whole cell RNAs but also those concentrated in the nucleus and cytosol. Long (>200 nt) and short RNAs (<200 nt) are being sequenced from each subcellular compartment, providing catalogs of potential miRNAs, snoRNA, promoter-associated short RNAs (PASRs) [27], and other short cellular RNAs. Total RNA from K562 and GM12878 cells has been mapped by hybridization to high-density tiling arrays and sequenced to a depth of >500 million paired-end 76 bp reads under conditions where the strand of the RNA transcript is determined, providing considerable depth of transcript coverage (see below).

**Figure 3:**
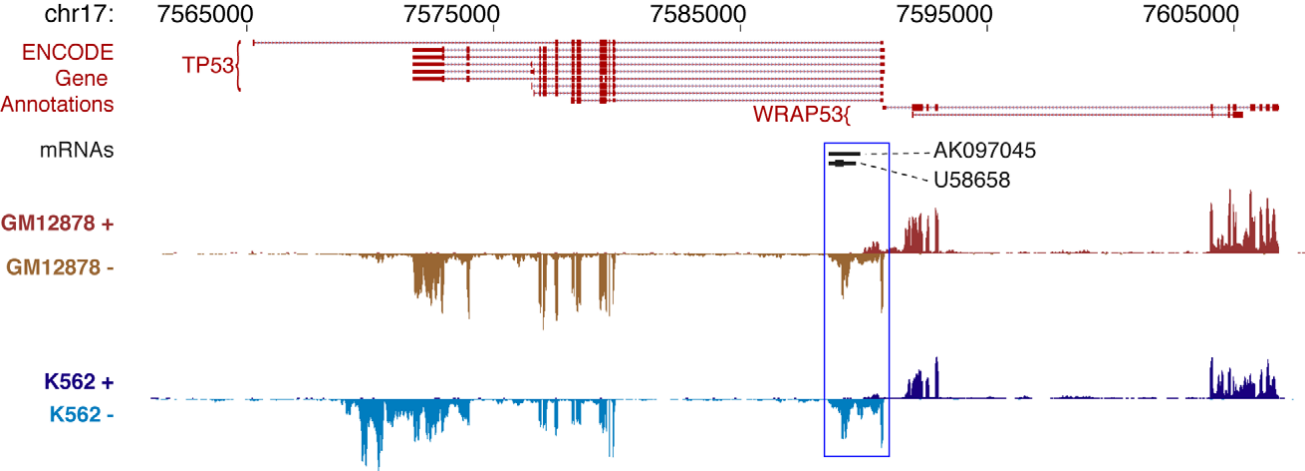
ENCODE gene and transcript annotations. The image shows selected ENCODE and other gene and transcript annotations in the region of the human *TP53* gene (region chr17:7,560,001–7,610,000 from the Human February 2009 (GRCh37/hg19) genome assembly). The annotated isoforms of *TP53* RNAs listed from the ENCODE Gene Annotations (GENCODE) are shown in the top tracks of the figure, along with annotation of the neighboring *WRAP53* gene. In black are two mRNA transcripts (U58658/AK097045) from GenBank. The bottom two tracks show the structure of the *TP53* region transcripts detected in nuclear polyadenylated poly A+ RNAs isolated from GM12878 and K562 cells. The RNA is characterized by RNA-seq and the RNAs detected are displayed according to the strand of origin (i.e. + and −). Signals are scaled and are present at each of the detected p53 exons. Signals are also evident at the U58658 [120] and AK097045 [121] regions located in the first 10 kb intron of the p53 gene (D17S2179E). The U58658/AK097045 transcripts are reported to be induced during differentiation of myeloid leukemia cells but are seen in both GM12878 and K562 cell lines. Finally the p53 isoform observed in K562 cells has a longer 3′UTR region than the isoform seen in the GM12878 cell line.

These analyses reveal that the human genome encodes a diverse array of transcripts. For example, in the proto-oncogene *TP53* locus, RNA-seq data indicate that, while *TP53* transcripts are accurately assigned to the minus strand, those for the oppositely transcribed, adjacent gene *WRAP53* emanate from the plus strand (Figure 3). An independent transcript within the first intron of *TP53* is also observed in both GM12878 and K562 cells (Figure 3).

Additional transcript annotations include exonic regions and splice junctions, transcription start sites (TSSs), transcript 3′ ends, spliced RNA length, locations of polyadenylation sites, and locations with direct evidence of protein expression. TSSs and 3′ ends of transcripts are being determined with two approaches, Paired-End diTag (PET) [28] and Cap-Analysis of Gene Expression (CAGE) [29]–[31] sequencing.

Transcript annotations throughout the genome are further corroborated by comparing tiling array data with deep sequencing data and by the manual curation described above. Additionally, selected compartment-specific RNA transcripts that cannot be mapped to the current build of the human genome sequence have been evaluated by 5′/3′ Rapid Amplification of cDNA Ends (RACE) [32], followed by RT-PCR cloning and sequencing. To assess putative protein products generated from novel RNA transcripts and isoforms, proteins may be sequenced and quantified by mass spectrometry and mapped back to their encoding transcripts [33],[34]. ENCODE has recently begun to study proteins from distinct subcellular compartments of K562 and GM12878 cells by using this complementary approach.

### *Cis*-Regulatory Regions

*Cis*-regulatory regions include diverse functional elements (e.g., promoters, enhancers, silencers, and insulators) that collectively modulate the magnitude, timing, and cell-specificity of gene expression [35]. The ENCODE Project is using multiple approaches to identify *cis*-regulatory regions, including localizing their characteristic chromatin signatures and identifying sites of occupancy of sequence-specific transcription factors. These approaches are being combined to create a comprehensive map of human *cis*-regulatory regions.

#### Chromatin structure and modification

Human *cis*-regulatory regions characteristically exhibit nuclease hypersensitivity [36]–[39] and may show increased solubility after chromatin fixation and fragmentation [40],[41]. Additionally, specific patterns of post-translational histone modifications [42],[43] have been connected with distinct classes of regions such as promoters and enhancers [3],[44]–[47] as well as regions subject to programmed repression by Polycomb complexes [48],[49] or other mechanisms [46],[50],[51]. Chromatin accessibility and histone modifications thus provide independent and complementary annotations of human regulatory DNA, and massively parallel, high-throughput DNA sequencing methods are being used by ENCODE to map these features on a genome-wide scale (Figure 2 and Table 1).

**Figure 4:**
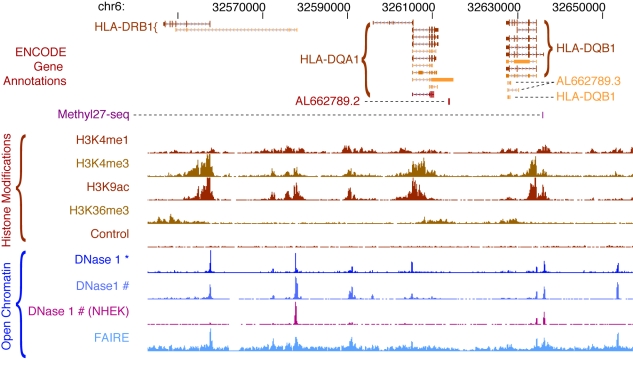
ENCODE chromatin annotations in the *HLA* locus. Chromatin features in a human lymphoblastoid cell line, GM12878, are displayed for a 114 kb region in the *HLA* locus. The top track shows the structures of the annotated isoforms of the *HLA-DRB1*, *HLA-DQA1*, and *HLA-DQB1* genes from the ENCODE Gene Annotations (GENCODE), revealing complex patterns of alternative splicing and several non-protein-coding transcripts overlapping the protein-coding transcripts. The purple mark on the next line shows that a CpG in the promoter of the *HLA-DQB1* gene is partially methylated (assayed on the Illumina Methylation27 BeadArray platform). The densities of four histone modifications associated with transcriptionally active loci are plotted next, along with the input control signal (generated by sequencing an aliquot of the sheared chromatin for which no immunoprecipitation was performed). The last lines plot the accessibility of DNA in chromatin to nucleases (DNaseI) and reduced coverage by nucleosomes (FAIRE); peaks on these lines are DNaseI hypersensitive sites. Note that the ENCODE Consortium generates DNaseI accessibility data by two alternative protocols marked by * and #. The magenta track shows DNaseI sensitivity in a different cell line, NHEK, for comparison.

DNaseI hypersensitive sites (DHSs) are being mapped by two techniques: (i) capture of free DNA ends at in vivo DNaseI cleavage sites with biotinylated adapters, followed by digestion with a TypeIIS restriction enzyme to generate ∼20 bp DNaseI cleavage site tags [52],[53] and (ii) direct sequencing of DNaseI cleavage sites at the ends of small (<300 bp) DNA fragments released by limiting treatment with DNaseI [54]–[56]. Chromatin structure is also being profiled with the FAIRE technique [40],[57],[58], in which chromatin from formaldehyde-crosslinked cells is sonicated in a fashion similar to ChIP and then extracted with phenol, followed by sequencing of soluble DNA fragments. An expanding panel of histone modifications (Figure 2) is being profiled by ChIP-seq [59]–[62]. In this method, chromatin from crosslinked cells is immunoprecipitated with antibodies to chromatin modifications (or other proteins of interest), the associated DNA is recovered, and the ends are subjected to massively parallel DNA sequencing. Control immunoprecipitations with a control IgG antibody or “input” chromatin—sonicated crosslinked chromatin that is not subjected to immune enrichment—are also sequenced for each cell type. These provide critical controls, as shearing of crosslinked chromatin may occur preferentially within certain regulatory DNA regions, typically promoters [41]. ENCODE chromatin data types are illustrated for a typical locus in Figure 4, which depicts the patterns of chromatin accessibility, DNaseI hypersensitive sites, and selected histone modifications in GM12878 cells.

For each chromatin data type, the “raw signal” is presented as the density of uniquely aligning sequence reads within 150 bp sliding windows in the human genome. In addition, some data are available as processed signal tracks in which filtering algorithms have been applied to reduce experimental noise. A variety of specialized statistical algorithms are applied to generate discrete high-confidence genomic annotations, including DHSs, broader regions of increased sensitivity to DNaseI, regions of enrichment by FAIRE, and regions with significant levels of specific histone modifications (see Tables 3 and S1). Notably, different histone modifications exhibit characteristic genomic distributions that may be either discrete (e.g., H3K4me3 over a promoter) or broad (e.g., H3K36me3 over an entire transcribed gene body). Because statistical false discovery rate (FDR) thresholds are applied to discrete annotations, the number of regions or elements identified under each assay type depends upon the threshold chosen. Optimal thresholds for an assay are typically determined by comparison to an independent and standard assay method or through reproducibility measurements (see below). Extensive validation of the detection of DNaseI hypersensitive sites is being performed independently with traditional Southern blotting, and more than 6,000 Southern images covering 224 regions in >12 cell types are available through the UCSC browser.

**Table 3:** Analysis tools applied by the ENCODE Consortium.

Class of Software	Description of Task	Examples^a^
Short read alignment	Computationally efficient alignment of short reads to the genome sequence	Bowtie, BWA, Maq, TopHat, GEM, STAR
Peak calling	Converting tag density to defined regions that show statistical properties consistent with binding activity	SPP, PeakSeq, Fseq, MACS, HotSpot
RNA processing	Processing RNA reads into exons and transcripts, with consideration of alternative splicing	Cufflinks, ERANGE, Fluxcapacitor
Integrative peak calling and classification	Jointly considering multiple assay signals to both define the location and character of different genomic regions	ChromHMM, Segway
Statistical tools for specific genomic tasks	Statistical methods developed for replicate-based thresholding, genome-wide-based overlap, and genome-based aggregation	IDR, GSC, ACT
Motif finding tools	Discovering the presence of sequence motifs in enriched peaks	MEME, Weeder
Data analysis frameworks	General frameworks to allow manipulation, comparison, and statistical analysis	R, Bioconductor, MatLab, Galaxy, DART, Genometools
Assign TFBS peaks to genes	Match TFBS to genes they are likely to regulate	GREAT
Compare TF binding and gene expression	Compare binding and expression; compare expressed versus nonexpressed genes	GenPattern, GSEA, Dchip
Conservation	Evaluates conservation of sequences across a range of species	phastCons, GERP, SCONE
Gene Ontology Analysis	Determine types of genes enriched for a given dataset	GO miner, BINGO, AmiGO
Network analysis	Examine relationships between genes	Cytoscape

a For full listings and references, see Table S1.

#### Transcription factor and RNA polymerase occupancy

Much of human gene regulation is determined by the binding of transcriptional regulatory proteins to their cognate sequence elements in *cis*-regulatory regions. ChIP-seq enables genome-scale mapping of transcription factor (TF) occupancy patterns in vivo [59],[60],[62] and is being extensively applied by ENCODE to create an atlas of regulatory factor binding in diverse cell types. ChIP-seq experiments rely on highly specific antibodies that are extensively characterized by immunoblot analysis and other criteria according to ENCODE experimental standards. High-quality antibodies are currently available for only a fraction of human TFs, and identifying suitable immunoreagents has been a major activity of ENCODE TF mapping groups. Alternative technologies, such as epitope tagging of TFs in their native genomic context using recombineering [63],[64], are also being explored.

**Figure 5:**
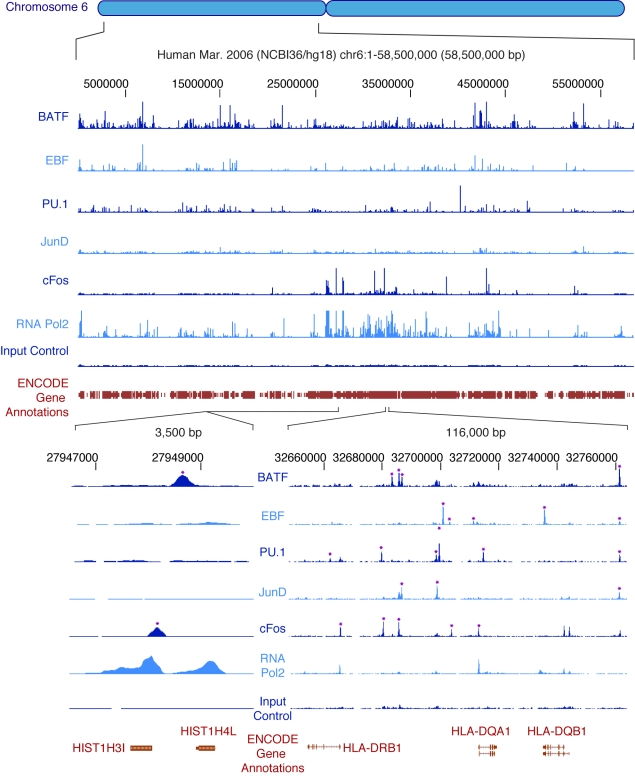
Occupancy of transcription factors and RNA polymerase 2 on human chromosome 6p as determined by ChIP-seq. The upper portion shows the ChIP-seq signal of five sequence-specific transcription factors and RNA Pol2 throughout the 58.5 Mb of the short arm of human chromosome 6 of the human lymphoblastoid cell line GM12878. Input control signal is shown below the RNA Pol2 data. At this level of resolution, the sites of strongest signal appear as vertical spikes in blue next to the name of each experiment (“BATF,” “EBF,” etc.). More detail can be seen in the bottom right portion, where a 116 kb segment of the HLA region is expanded; here, individual sites of occupancy can be seen mapping to specific regions of the three HLA genes shown at the bottom, with asterisks indicating binding sites called by peak calling software. Finally, the lower left region shows a 3,500 bp region around two tandem histone genes, with RNA Pol2 occupancy at both promoters and two of the five transcription factors, BATF and cFos, occupying sites nearby. Selected annotations from the ENCODE Gene Annotations are shown in each case.

ENCODE has applied ChIP-seq to create occupancy maps for a variety of TFs, RNA polymerase 2 (RNA Pol2) including both unphosphorylated (initiating) and phosphorylated (elongating) forms, and RNA polymerase 3 (RNA Pol3). The localization patterns of five transcription factors and RNA Pol2 in GM12878 lymphoblastoid cells are shown for a typical locus in Figure 5. Sequence reads are processed as described above for DNaseI, FAIRE, and histone modification experiments, including the application of specialized peak-calling algorithms that use input chromatin or control immunoprecipitation data to identify potential false-positives introduced by sonication or sequencing biases (Table 3). Although different peak-callers vary in performance, the strongest peaks are generally identified by multiple algorithms. Most of the sites identified by ChIP-seq are also detected by traditional ChIP-qPCR [65] or are consistent with sites reported in the literature. For example, 98% of 112 sites of CTCF occupancy previously identified by using both ChIP-chip and ChIP-qPCR [66] are also identified in ENCODE CTCF data. Whereas the binding of sequence-specific TFs is typically highly localized resulting in tight sequence tag peaks, signal from antibodies that recognize the phosphorylated (elongating) form of RNA Pol2 may detect occupancy over a wide region encompassing both the site of transcription initiation as well as the domain of elongation. Comparisons among ENCODE groups have revealed that TF and RNA Pol2 occupancy maps generated independently by different groups are highly consistent.

### Additional Data Types

ENCODE is also generating additional data types to complement production projects and benchmark novel technologies. An overview of these datasets is provided in Table 1.

#### DNA methylation

In vertebrate genomes, methylation at position 5 of the cytosine in CpG dinucleotides is a heritable “epigenetic” mark that has been connected with both transcriptional silencing and imprinting [67],[68]. ENCODE is applying several complementary approaches to measure DNA methylation. All ENCODE cell types are being assayed using two direct methods for measuring DNA methylation following sodium bisulfite conversion, which enables quantitative analysis of methylcytosines: interrogation of the methylation status of 27,000 CpGs with the Illumina Methyl27 assay [69]–[72] and Reduced Representation Bisulfite Sequencing (RRBS) [73], which couples *Msp*I restriction enzyme digestion, size selection, bisulfite treatment, and sequencing to interrogate the methylation status of >1,000,000 CpGs largely concentrated within promoter regions and CpG islands. Data from an indirect approach using a methylation-sensitive restriction enzyme (Methyl-seq) [74] are also available for a subset of cell types. These three approaches measure DNA methylation in defined (though overlapping) subsets of the human genome and provide quantitative determinations of the fraction of CpG methylation at each site.

#### DNaseI footprints

DNaseI footprinting [75] enables visualization of regulatory factor occupancy on DNA in vivo at nucleotide resolution and has been widely applied to delineate the fine structure of *cis-*regulatory regions [76]. Deep sampling of highly enriched libraries of DNaseI-released fragments (see above) enables digital quantification of per nucleotide DNaseI cleavage, which in turn enables resolution of DNaseI footprints on a large scale [55],[77],[78]. Digital genomic footprinting is being applied on a large scale within ENCODE to identify millions of DNaseI footprints across >12 cell types, many of which localize the specific cognate regulatory motifs for factors profiled by ChIP-seq.

#### Sequence and structural variation

Genotypic and structural variations within all ENCODE cell types are being interrogated at ∼1 million positions distributed approximately every 1.5 kb along the human genome, providing a finely grained map of allelic variation and sequence copy number gains and losses. Genotyping data are generated with the Illumina Infinium platform [79], and the results are reported as genotypes and as intensity value ratios for each allele. The genotype and sequence data from GM12878 generated by the 1,000 Genomes Project are being integrated with sequence data from ENCODE chromatin, transcription, TF occupancy, DNA methylation, and other assays to facilitate recognition of functional allelic variation, a significant contributor to phenotypic variability in gene expression [80],[81]. The data also permit determination of the sequence copy number gains and losses found in every human genome [82]–[84], which are particularly prevalent in cell lines of malignant origin.

#### Long-range Chromatin interactions

Because *cis*-regulatory elements such as enhancers can control genes from distances of tens to hundreds of kb through looping interactions [85], a major challenge presented by ENCODE data is to connect distal regulatory elements with their cognate promoter(s). To map this connectivity, the Consortium is applying the 5C method [86], an enhanced version of Chromosome Conformation Capture (3C) [87], to selected cell lines. 5C has been applied comprehensively to the ENCODE pilot regions as well as to map the interactions between distal DNaseI hypersensitive sites and transcriptional start sites across chromosome 21 and selected domains throughout the genome. Special interfaces have been developed to visualize these 3-dimensional genomic data and are publicly available at http://my5C.umassmed.edu [88].

#### Protein:RNA interactions

RNA-binding proteins play a major role in regulating gene expression through control of mRNA translation, stability, and/or localization. Occupancy of RNA-binding proteins (RBPs) on RNA can be determined by using immunoprecipitation-based approaches (RIP-chip and RIP-seq) [89]–[92] analogous to those used for measuring TF occupancy. To generate maps of RBP∶RNA associations and binding sites, a combination of RIP-chip and RIP-seq are being used. These approaches are currently targeting 4–6 RBPs in five human cell types (K562, GM12878, H1 ES, HeLa, and HepG2). RBP associations with non-coding RNA and with mRNA are also being explored.

#### Identification of functional elements with integrative analysis and fine-scale assays of biochemical elements

ChIP-seq of TFs and chromatin modifications may identify genomic regions bound by transcription factors in living cells but do not reveal which segments bound by a given TF are functionally important for transcription. By applying integrative approaches that incorporate histone modifications typical of enhancers (e.g., histone H3, Lysine 4 monomethylation), promoters (e.g., histone H3, Lysine 4 trimethylation), and silencers (e.g., Histone H3, Lysine 27, and Lysine 9 trimethylation), ENCODE is categorizing putative functional elements and testing a subset for activities in the context of transient transfection/reporter gene assays [93]–[97]. To further pinpoint the biological activities associated with specific regions of TF binding and chromatin modification within promoters, hundreds of TF binding sites have been mutagenized, and the mutant promoters are being assayed for effects on reporter gene transcription by transient transfection assays. This approach is enabling identification of specific TF binding sites that lead to activation and others associated with transcriptional repression.

#### Proteomics

To assess putative protein products generated from novel RNA transcripts and isoforms, proteins are sequenced and quantified by mass spectrometry and mapped back to their encoding transcripts [33],[34],[98]. ENCODE has recently begun to study proteins from distinct subcellular compartments of K562 and GM12878 with this complementary approach.

#### Evolutionary conservation

Evolutionary conservation is an important indicator of biological function. ENCODE is approaching evolutionary analysis from two directions. Functional properties are being assigned to conserved sequence elements identified through multi-species alignments, and conversely, the evolutionary histories of biochemically defined elements are being deduced. Multiple alignments of the genomes of 33 mammalian species have been constructed by using the Enredo, Pecan, Ortheus approach (EPO) [99],[100], and complementary multiple alignments are available through the UCSC browser (UCSC Lastz/ChainNet/Multiz). These alignments enable measurement of evolutionary constraint at single-nucleotide resolution using GERP [101], SCONE [102], PhyloP [103], and other algorithms. In addition, conservation of DNA secondary structure based on hydroxyl radical cleavage patterns is being analyzed with the Chai algorithm [7].

### Data Production Standards and Assessment of Data Quality

With the aim of ensuring quality and consistency, ENCODE has defined standards for collecting and processing each data type. These standards encompass all major experimental components, including cell growth conditions, antibody characterization, requirements for controls and biological replicates, and assessment of reproducibility. Standard formats for data submission are used that capture all relevant data parameters and experimental conditions, and these are available at the public ENCODE portal (http://genome.ucsc.edu/ENCODE/dataStandards.html). All ENCODE data are reviewed by a dedicated quality assurance team at the Data Coordination Center before release to the public. Experiments are considered to be *verified* when two highly concordant biological replicates have been obtained with the same experimental technique. In addition, a key quality goal of ENCODE is to provide *validation* at multiple levels, which can be further buttressed by cross-correlation between disparate data types. For example, we routinely perform parallel analysis of the same biological samples with alternate detection technologies (for example, ChIP-seq versus ChIP-chip or ChIP-qPCR). We have also compared our genome-wide results to “gold-standard” data from individual locus studies, such as DNase-seq versus independently performed conventional (Southern-based) DNaseI hypersensitivity studies. Cross-correlation of independent but related ENCODE data types with one another, such as DNaseI hypersensitivity, FAIRE, transcription factor occupancy, and histone modification patterns, can provide added confidence in the identification of specific DNA elements. Similarly, cross-correlation between long RNA-seq, CAGE, and TAF1 ChIP-seq data can strengthen confidence in a candidate location for transcription initiation. Finally, ENCODE is performing pilot tests for the biological activity of DNA elements to the predictive potential of various ENCODE biochemical signatures for certain biological functions. Examples include transfection assays in cultured human cells and injection assays in fish embryos to test for enhancer, silencer, or insulator activities in DNA elements identified by binding of specific groups of TFs or the presence of DNaseI hypersensitive sites or certain chromatin marks. Ultimately, defining the full biological role of a DNA element in its native chromosomal location and organismic context is the greatest challenge. ENCODE is beginning to approach this by integrating its data with results from other studies of in situ knockouts and/or knockdowns, or the identification of specific naturally occurring single base mutations and small deletions associated with changes in gene expression. However, we expect that deep insights into the function of most elements will ultimately come from the community of biologists who will build on ENCODE data or use them to complement their own experiments.

### Current Scope and Completeness of ENCODE Data

A catalog of ENCODE datasets is available at http://encodeproject.org. These data provide evidence that ∼1 Gigabase (Gb; 32%) of the human genome sequence is represented in steady-state, predominantly processed RNA populations. We have also delineated more than 2 million potential regulatory DNA regions through chromatin and TF mapping studies.

**Figure 6:**
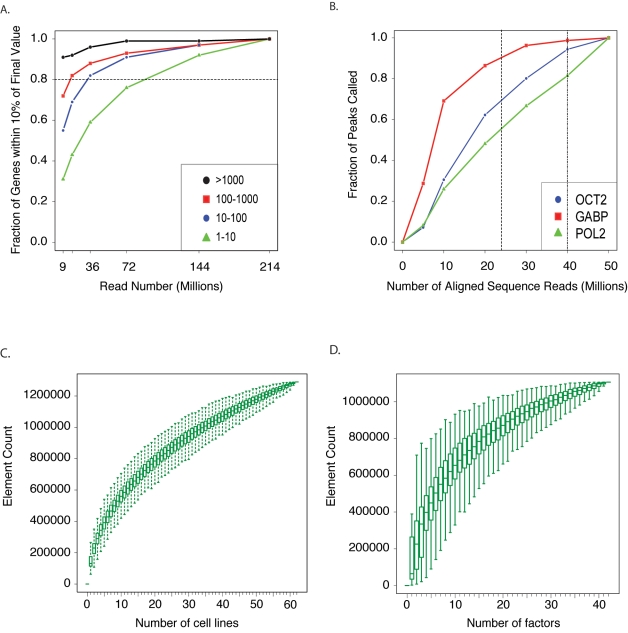
Incremental discovery of transcribed elements and regulatory DNA. (A) Robustness of gene expression quantification relative to sequencing depth. PolyA-selected RNA from H1 human embryonic stem cells was sequenced to 214 million mapped reads. The number of reads (indicated on the *x*-axis) was sampled from the total, and gene expression (in FPKM) was calculated and compared to the gene expression values resulting from all the reads (final values). Gene expression levels were split into four abundance classes and the fraction of genes in each class with RPKM values within 10% of the final values was calculated. At ∼80 million mapped reads, more than 80% of the low abundance class of genes is robustly quantified according to this measure (horizontal dotted line). Abundances for the classes in RPKM are given in the inset box. (B) Effect of number of reads on fractions of peaks called in ChIP-seq. ChIP-seq experiments for three sequence-specific transcription factors were sequenced to a depth of 50 million aligned reads. To evaluate the effect of read depth on the number of binding sites identified, peaks were called with the MACS algorithm at various read depths, and the fraction of the total number of peaks that were identified at each read depth are shown. For sequence-specific transcription factors that have strong signal with ChIP-seq, such as GABP, approximately 24 million reads (dashed vertical line) are sufficient to capture 90% of the binding sites. However, for more general sequence-specific factors (e.g., OCT2), additional sequencing continues to yield additional binding site information. RNA Pol2, which interacts with DNA broadly across genes, maintains a nearly linear gain in binding information through 50 million aligned reads. (C) Saturation analysis of ENCODE DNaseI hypersensitivity data with increasing numbers of cell lines. The plot shows the extent of saturation of DNaseI hypersensitivity sites (DHSs) discovered as increasing numbers of cell lines are studied. The plot is generated from the ENCODE DNaseI elements defined at the end of January 2010 (from http://hgdownload.cse.ucsc.edu/goldenPath/hg18/encodeDCC) as follows. We first define a set of DHSs from the overlap of all DHS data across all cell lines. Where overlapping elements are identified in two or more cell lines, these are determined to represent the same element and fused up to a maximum size of 5 kb. Elements above this limit are split and counted as distinct. We then calculate the subset of these elements represented by each single cell line experiment. The distribution of element counts for each single cell line is plotted as a box plot with the median at position 1 on the *x*-axis. We next calculate the element contributions of all possible pairs of cell line experiments and plot this distribution at position 2. We continue to do this for all incremental steps up to and including all cell lines (which is by definition only a single data point). (D) Saturation of TF ChIP-seq elements in K562 cells. This plot illustrates the saturation of elements identified by TF ChIP-seq as additional factors are analyzed within the same cell line. The plot is generated by the equivalent approach as described in (C), except the data are now the set of all elements defined by ChIP-seq analysis of K562 cells with 42 different transcription factors. The data were from the January 2010 data freeze from http://hgdownload.cse.ucsc.edu/goldenPath/hg18/encodeDCC. For consistency, the peak calls from all ChIP-seq data were generated by a uniform processing pipeline with the Peakseq peak caller and IDR replicate reconciliation.

The assessment of the completeness of detection of any given element is challenging. To analyze the detection of transcripts in a single experiment, we have sequenced to substantial depth and used a sampling approach to estimate the number of reads needed to approach complete sampling of the RNA population (Figure 6A) [104]. For example, analyzing RNA transcripts with about 80 million mapped reads yields robust quantification of more than 80% of the lowest abundance class of genes (2–19 reads per kilobase per million mapped tags, RPKM) [24]. Measuring RNAs across multiple cell types, we find that, after the analysis of seven cell lines, 68% of the GENCODE transcripts can be detected with RPKM >1.

In the case of regulatory DNA, we have analyzed the detection of regulatory DNA by using three approaches: 1) the saturation of occupancy site discovery for a single transcription factor within a single cell type as a function of sequencing read depth, 2) the incremental discovery of DNaseI hypersensitive sites or the occupancy sites for a single TF across multiple cell types, and 3) the incremental rate of collective TF occupancy site discovery for all TFs across multiple cell types.

For detecting TF binding sites by ChIP-seq, we have found that the number of significant binding sites increases as a function of sequencing depth and that this number varies widely by transcription factor. For example, as shown in Figure 6B, 90% of detectable sites for the transcription factor GABP can be identified by using the MACS peak calling program at a depth of 24 million reads, whereas only 55% of detectable RNA Pol2 sites are identified at this depth when an antibody that recognizes both initiating and elongating forms of the enzyme is used. Even at 50 million reads, the number of sites is not saturated for RNA Pol2 with this antibody. It is important to note that determinations of saturation may vary with the use of different antibodies and laboratory protocols. For instance, a different RNA Pol2 antibody that recognizes unphosphorylated, non-elongating RNA Pol2 bound only at promoters requires fewer reads to reach saturation [105]. For practical purposes, ENCODE currently uses a minimum sequencing depth of 20 M uniquely mapped reads for sequence-specific transcription factors. For data generated prior to June 1, 2010, this figure was 12 M.

To assess the incremental discovery of regulatory DNA across different cell types, it was necessary to account for the non-uniform correlation between cell lines and assays (see Figure 6C legend for details). We therefore examined all possible orderings of either cell types or assays and calculated the distribution of elements discovered as the number of cell types or assays increases, presented as saturation distribution plots (Figure 6C and 6D, respectively). For DNase hypersensitive sites, we observe a steady increase in the mean number of sites discovered as additional cell types are tested up to and including the 62 different cell types examined to date, indicating that new elements continue to be identified at a relatively high rate as additional cell types are sampled (Figure 6C). Analysis of CTCF sites across 28 cell types using this approach shows similar behavior. Analysis of binding sites for 42 TFs in the cell line with most data (K562) also shows that saturation of the binding sites for these factors has not yet been achieved. These results indicate that additional cell lines need to be analyzed for DNaseI and many transcription factors, and that many more transcription factors need to be analyzed within single cell types to capture all the regulatory information for a given factor across the genome. The implications of these trends for defining the extent of regulatory DNA within the human genome sequence is as yet unclear.

## III. Accessing ENCODE Data

### ENCODE Data Release and Use Policy

The ENCODE Data Release and Use Policy is described at http://www.encodeproject.org/ENCODE/terms.html. Briefly, ENCODE data are released for viewing in a publicly accessible browser (initially at http://genome-preview.ucsc.edu/ENCODE and, after additional quality checks, at http://encodeproject.org). The data are available for download and pre-publication analysis of any kind, as soon as they are verified (i.e., shown to be reproducible). However, consistent with the principles stated in the Toronto Genomic Data Use Agreement [106], the ENCODE Consortium data producers request that they have the first publication on genome-wide analyses of ENCODE data, within a 9-month timeline from its submission. The timeline for each dataset is clearly displayed in the information section for each dataset. This parallels policies of other large consortia, such as the HapMap Project (http://www.hapmap.org), that attempt to balance the goal of rapid data release with the ability of data producers to publish initial analyses of their work. Once a producer has published a dataset during this 9-month period, anyone may publish freely on the data. The embargo applies only to global analysis, and the ENCODE Consortium expects and encourages immediate use and publication of information at one or a few loci, without any consultation or permission. For such uses, identifying ENCODE as the source of the data by citing this article is requested.

### Public Repositories of ENCODE Data

After curation and review at the Data Coordination Center, all processed ENCODE data are publicly released to the UCSC Genome Browser database (http://genome.ucsc.edu). Accessioning of ENCODE data at the NCBI Gene Expression Omnibus (GEO; http://www.ncbi.nlm.nih.gov/geo/info/ENCODE.html) is underway. Primary DNA sequence reads are stored at UCSC and the NCBI Sequence Read Archive (SRA; http://www.ncbi.nlm.nih.gov/Traces/sra/sra.cgi?) and will also be retrievable via GEO. Primary data derived from DNA microarrays (for example, for gene expression) are deposited directly to GEO. The processed data are also formatted for viewing in the UCSC browser. Metadata, including information on antibodies, cell culture conditions, and other experimental parameters, are deposited into the UCSC database, as are results of validation experiments. Easy retrieval of ENCODE data to a user's desktop is facilitated by the UCSC Table Browser tool (http://genome.ucsc.edu/cgi-bin/hgTables?org=human), which does not require programming skills. Computationally sophisticated users may gain direct access to data through application programming interfaces (APIs) at both the UCSC browser and NCBI and by downloading files from http://genome.ucsc.edu/ENCODE/downloads.html.

An overview of ENCODE data types and the location of the data repository for each type is presented in Table 4.

**Table 4:** Overview of ENCODE data types.

Data	Description	Location
Metadata	Experimental parameters (e.g., growth conditions, antibody characterization)	UCSC, GEO
Primary data images	CCD camera images from sequencers or microarrays	Not archived
Sequence reads/microarray signal	Minimally processed experimental data; reads and quality information; probe locations and intensities	UCSC, GEO, SRA
Aligned sequence reads	Sequence reads and genomic positions	UCSC, GEO
Genomic signal	Sequence tag density (sliding window); cumulative base coverage or density by sequencing or read pseudo-extension; microarray probe intensity	UCSC, GEO
Enriched region calls/scores/*p* or *q* values	Putative binding or transcribed regions	UCSC, GEO

## IV. Working with ENCODE Data

### Using ENCODE Data in the UCSC Browser

**Figure 7:**
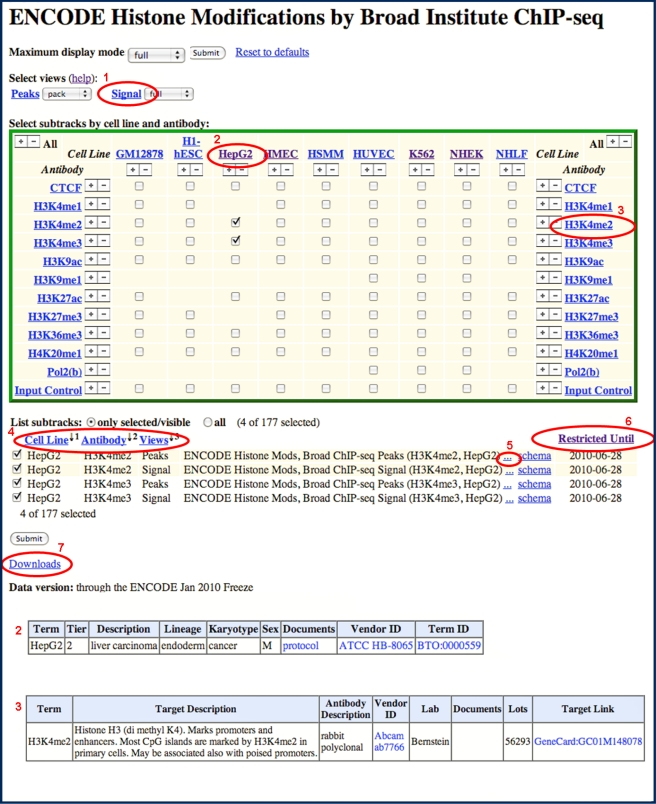
Accessing ENCODE data at the UCSC Portal. Data and results for the ENCODE Project are accessible at the UCSC portal (http://genome.ucsc.edu/ENCODE). “Signal tracks” for the different datasets are selected and displayed in the genome browser to generate images such as those shown in Figures 3–4. The datasets are available from the Track Settings page; an example is shown that illustrates some of the key controls. A dataset is selected and the Signal display plots the values of an assay for a given feature more or less continuously along a chromosome. The height, range for the *y*-axis, windowing function, and many other aspects of the graph are controlled in the Signal Configuration window, accessed by clicking on “Signal” (red oval #1). ENCODE data are commonly generated on multiple cell lines; information about each can be accessed by clicking on the name of the cell line or antibody (e.g., HepG2, red oval #2). Many ENCODE tracks are actually composites of multiple subtracks; these can be turned on and off by using the boxes in the central matrix or in the subtrack list below. Subtracks can be reordered individually by using drag and drop in the browser image or the Track Settings page, or in logical groups by using the “Cell/Antibody/Views” (red oval #4) ordering controls. Additional information about the feature and the assay, such as the antibody used, can be obtained by clicking on the name of the feature. Some restrictions to the use of ENCODE data apply for a 9-month period after deposit of the data; the end of that 9-month period is given by the “Restricted Until” date. Full data can be downloaded by clicking on the “Downloads” link (red oval #7).

Many users will want to view and interpret the ENCODE data for particular genes of interest. At the online ENCODE portal (http://encodeproject.org), users should follow a “Genome Browser” link to visualize the data in the context of other genome annotations. Currently, it is useful for users to examine both the hg18 and the hg19 genome browsers. The hg18 has the ENCODE Integrated Regulation Track on by default, which shows a huge amount of data in a small amount of space. The hg19 browser has newer datasets, and more ENCODE data than are available on hg18. Work is in progress to remap the older hg18 datasets to hg19 and generate integrated ENCODE tracks. On either browser, additional ENCODE tracks are marked by a double helix logo in the browser track groups for genes, transcripts, and regulatory features. Users can turn tracks on or off to develop the views most useful to them (Figure 7). To aid users in navigating the rich variety of data tracks, the ENCODE portal also provides a detailed online tutorial that covers data display, data download, and analysis functions available through the browser. Examples applying ENCODE data at individual loci to specific biological or medical issues are a good starting point for exploration and use of the data. Thus, we also provide a collection of examples at the “session gallery” at the ENCODE portal. Users are encouraged to submit additional examples; we anticipate that this community-based sharing of insights will accelerate the use and impact of the ENCODE data.

### An Illustrative Example

Numerous genome-wide association studies (GWAS) that link human genome sequence variants with the risk of disease or with common quantitative phenotypes have now become available. However, in most cases, the molecular consequences of disease- or trait-associated variants for human physiology are not understood [107]. In more than 400 studies compiled in the GWAS catalog [108], only a small minority of the trait/disease-associated SNPs (TASs) occur in protein-coding regions; the large majority (89%) are in noncoding regions. We therefore expect that the accumulating functional annotation of the genome by ENCODE will contribute substantially to functional interpretation of these TASs.

**Figure 8:**
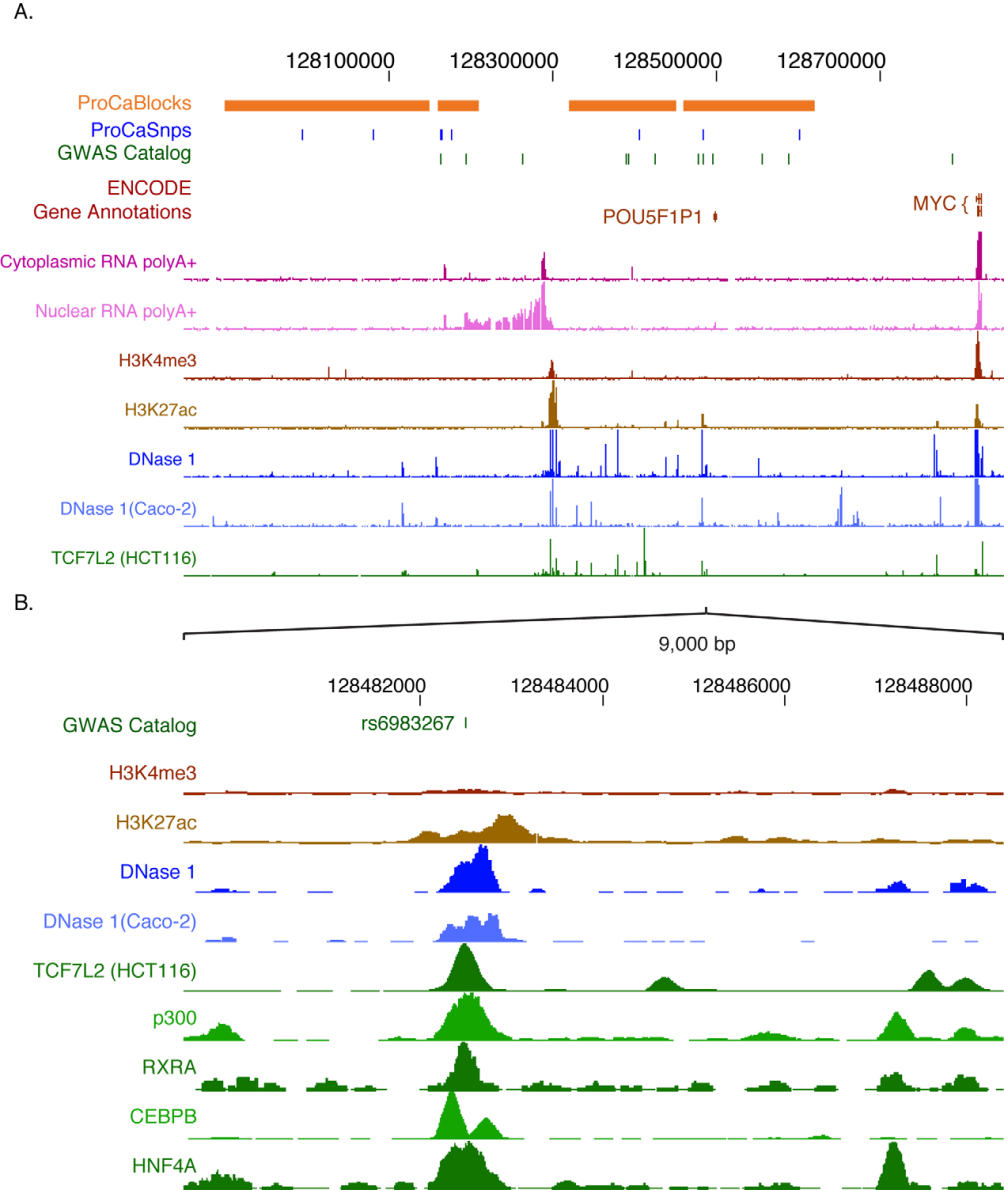
ENCODE data indicate non-coding regions in the human chromosome 8q24 loci associated with cancer. (A) A 1 Mb region including *MYC* and a gene desert upstream shows the linkage disequilibrium blocks and positions of SNPs associated with breast and prostate cancer, with both a custom track based on [121] and the resident track from the GWAS catalog. ENCODE tracks include GENCODE gene annotations, results of mapping RNAs to high-density Affymetrix tiling arrays (cytoplasmic and nuclear polyA+ RNA), mapping of histone modifications (H3K4me3 and H3K27Ac), DNaseI hypersensitive sites in liver and colon carcinoma cell lines (HepG2 and Caco-2), and occupancy by the transcription factor TCF7L2 in HCT116 cells. (B) Expanded view of a 9 kb region containing the cancer-associated SNP *rs6983267* (shown on the top line). In addition to the histone modifications, DNaseI hypersensitive sites and factor occupancy described in (A), the ENCODE tracks also show occupancy by the coactivator p300 and the transcription factors RXRA, CEBPB, and HNF4A. Except as otherwise noted in brackets, the ENCODE data shown here are from the liver carcinoma cell line HepG2.

For example, common variants within a ∼1 Mb region upstream of the c-Myc proto-oncogene at 8q24 have been associated with cancers of the colon, prostate, and breast (Figure 8A) [109]–[111]. ENCODE data on transcripts, histone modifications, DNase hypersensitive sites, and TF occupancy show strong, localized signals in the vicinity of major cancer-associated SNPs. One variant (*rs698327*) lies within a DNase hypersensitive site that is bound by several TFs and the enhancer-associated protein p300 and contains histone modification patterns typical of enhancers (high H3K4me1, low H3K4me3; Figure 8B). Recent studies have shown enhancer activity and allele-specific binding of TCF7L2 at this site [112], with the risk allele showing greater binding and activity [113],[114]. Moreover, this element appears to contact the downstream c-Myc gene in vivo, compatible with enhancer function [114],[115]. Similarly, several regions predicted via ENCODE data to be involved in gene regulation are close to SNPs in the *BCL11A* gene associated with persistent expression of fetal hemoglobin (Figure S2). These examples show that the simple overlay of ENCODE data with candidate non-coding risk-associated variants may readily identify specific genomic elements as leading candidates for investigation as probable effectors of phenotypic effects via alterations in gene expression or other genomic regulatory processes. Importantly, even data from cell types not directly associated with the phenotype of interest may be of considerable value for hypothesis generation. It is reasonable to expect that application of current and future ENCODE data will provide useful information concerning the mechanism(s) whereby genomic variation influences susceptibility to disease, which then can then be tested experimentally.

### Limitations of ENCODE Annotations

All ENCODE datasets to date are from populations of cells. Therefore, the resulting data integrate over the entire cell population, which may be physiologically and genetically inhomogeneous. Thus, the source cell cultures in the ENCODE experiments are not typically synchronized with respect to the cell cycle and, as with all such samples, local micro-environments in culture may also vary, leading to physiological differences in cell state within each culture. In addition, one Tier 1 cell line (K562) and two Tier 2 cell lines (HepG2 and HeLa) are known to have abnormal genomes and karyotypes, with genome instability. Finally, some future Tier 3 tissue samples or primary cultures may be inherently heterogeneous in cell type composition. Averaging over heterogeneity in physiology and/or genotype produces an amalgamation of the contributing patterns of gene expression, factor occupancy, and chromatin status that must be considered when using the data. Future improvements in genome-wide methodology that allow the use of much smaller amounts of primary samples, or follow-up experiments in single cells when possible, may allow us to overcome many of these caveats.

The use of DNA sequencing to annotate functional genomic features is constrained by the ability to place short sequence reads accurately within the human genome sequence. Most ENCODE data types currently represented in the UCSC browser use only those sequence reads that map uniquely to the genome. Thus, centromeric and telomeric segments (collectively ∼15% of the genome and enriched in recent transposon insertions and segmental duplications) as well as sequences not present in the current genome sequence build [116] are not subject to reliable annotation by our current techniques. However, such information can be gleaned through mining of the publicly available raw sequence read datasets generated by ENCODE.

It is useful to recognize that the confidence with which different classes of ENCODE elements can be related to a candidate function varies. For example, ENCODE can identify with high confidence new internal exons of protein-coding genes, based on RNA-seq data for long polyA+ RNA. Other features, such as candidate promoters, can be identified with less, yet still good, confidence by combining data from RNA-seq, CAGE-tags, and RNA polymerase 2 (RNA Pol2) and TAF1 occupancy. Still other ENCODE biochemical signatures come with much lower confidence about function, such as a candidate transcriptional enhancer supported by ChIP-seq evidence for binding of a single transcription factor.

Identification of genomic regions enriched by ENCODE biochemical assays relies on the application of statistical analyses and the selection of threshold significance levels, which may vary between the algorithms used for particular data types. Accordingly, discrete annotations, such as TF occupancy or DNaseI hypersensitive sites, should be considered in the context of reported *p* values, *q* values, or false discovery rates, which are conservative in many cases. For data types that lack focal enrichment, such as certain histone modifications and many RNA Pol2-bound regions, broad segments of significant enrichment have been delineated that encompass considerable quantitative variation in the signal strength along the genome.

## V. ENCODE Data Analysis

Development and implementation of algorithms and pipelines for processing and analyzing data has been a major activity of the ENCODE Project. Because massively parallel DNA sequencing has been the main type of data generated by the Consortium, much of the algorithmic development and data analysis to date has been concerned with issues related to producing and interpreting such data. Software packages and algorithms commonly used in the ENCODE Consortium are summarized in Tables 3 and S1.

In general, the analysis of sequencing-based measurements of functional or biochemical genomic parameters proceeds through three major phases. In the first phase, the short sequences that are the output of the experimental method are aligned to the reference genome. Algorithm development for efficient and accurate alignment of short read sequences to the human genome is a rapidly developing field, and ENCODE groups employ a variety of the state-of-the-art software (see Tables 3 and S1). In the second phase, the initial sequence mapping is processed to identify significantly enriched regions from the read density. For ChIP-seq (TFs and histone modification), DNase-seq or FAIRE-seq, both highly localized peaks or broader enriched regions may be identified. Within the ENCODE Consortium, each data production group provides lists of enriched regions or elements within their own data, which are available through the ENCODE portal. It should be noted that, for most data types, the majority of enriched regions show relatively weak absolute signal, necessitating the application of conservative statistical thresholds. For some data, such as those derived from sampling RNA species (e.g., RNA-seq), additional algorithms and processing are used to handle transcript structures and the recognition of splicing events.

The final stage of analysis involves integrating the identified regions of enriched signal with each other and with other data types. An important prerequisite to data integration is the availability of uniformly processed datasets. Therefore, in addition to the processing pipelines developed by individual production groups, ENCODE has devoted considerable effort toward establishing robust uniform processing for phases 1 and 2 to enable integration. For signal comparison, specific consideration has been given to deriving a normalized view of the sequence read density of each experiment. In the case of ChIP-seq for TFs, this process includes in silico extension of the sequence alignment to reflect the experimentally determined average lengths of the input DNA molecules that are sampled by the short sequence tag, compensation for repetitive sequences that may lead to alignment with multiple genomic locations, and consideration of the read density of the relevant control or input chromatin experiment. ENCODE has adopted a uniform standardized peak-calling approach for transcription factor ChIP-seq, including a robust and conservative replicate reconciliation statistic (Irreproducible Discovery Rate, IDR [117], to yield comparable consensus peak calls. As the project continues, we expect further standardizations to be developed.

There are many different ways to analyze and integrate large, diverse datasets. Some of the basic approaches include assigning features to existing annotations (e.g., assigning transcribed regions to annotated genes or Pol2-binding peaks to likely genes), discovery of correlations among features, and identification of particular gene classes (e.g., Gene Ontology categories) preferentially highlighted by a given annotation. Many software tools exist in the community for these purposes, including some developed within the ENCODE Project, such as the Genome Structure Correction statistic for assessing overlap significance [3]. Software tools used for integration by ENCODE are summarized in Tables 3 and S1.

## VI. Future Plans and Challenges

### Data Production Plans

The challenge of achieving complete coverage of all functional elements in the human genome is substantial. The adult human body contains several hundred distinct cell types, each of which expresses a unique subset of the ∼1,500 TFs encoded in the human genome [118]. Furthermore, the brain alone contains thousands of types of neurons that are likely to express not only different sets of TFs but also a larger variety of non-coding RNAs [119]. In addition, each cell type may exhibit a diverse array of responses to exogenous stimuli such as environmental conditions or chemical agents. Broad areas of fundamental chromosome function, such as meiosis and recombination, remain unexplored. Furthermore, ENCODE has focused chiefly on definitive cells and cell lines, bypassing the substantial complexity of development and differentiation. A truly comprehensive atlas of human functional elements is not practical with current technologies, motivating our focus on performing the available assays in a range of cell types that will provide substantial near-term utility. ENCODE is currently developing a strategy for addressing this cellular space in a timely manner that maximizes the value to the scientific community. Feedback from the user community will be a critical component of this process.

### Integrating ENCODE with Other Projects and the Scientific Community

To understand better and functionally annotate the human genome, ENCODE is making efforts to analyze and integrate data within the project and with other large-scale projects. These efforts include 1) defining promoter and enhancer regions by combining transcript mapping and biochemical marks, 2) delineating distinct classes of regions within the genomic landscape by their specific combinations of biochemical and functional characteristics, and 3) defining transcription factor co-associations and regulatory networks. These efforts aim to extend our understanding of the functions of the different biochemical elements in gene regulation and gene expression.

One of the major motivations for the ENCODE Project has been to aid in the interpretation of human genome variation that is associated with disease or quantitative phenotypes. The Consortium is therefore working to combine ENCODE data with those from other large-scale studies, including the 1,000 Genomes Project, to study, for example, how SNPs and structural variation may affect transcript, regulatory, and DNA methylation data. We foresee a time in the near future when the biochemical features defined by ENCODE are routinely combined with GWAS and other sequence variation–driven studies of human phenotypes. Analogously, the systematic profiling of epigenomic features across ex vivo tissues and stem cells currently being undertaken by the NIH Roadmap Epigenomics program will provide synergistic data and the opportunity to observe the state and behavior of ENCODE-identified elements in human tissues representing healthy and disease states.

These are but a few of many applications of the ENCODE data. Investigators focused on one or a few genes should find many new insights within the ENCODE data. Indeed, these investigators are in the best position to infer potential functions and mechanisms from the ENCODE data—ones that will also lead to testable hypotheses. Thus, we expect that the work of many investigators will be enhanced by these data and that their results will in turn inform the development of the project going forward.

Finally, we also expect that comprehensive paradigms for gene regulation will begin to emerge from our work and similar work from many laboratories. Deciphering the “regulatory code” within the genome and its associated epigenetic signals is a grand and complex challenge. The data contributed by ENCODE in conjunction with complementary efforts will be foundational to this effort, but equally important will be novel methods for genome-wide analysis, model building, and hypothesis testing. We therefore expect the ENCODE Project to be a major contributor not only of data but also novel technologies for deciphering the human genome and those of other organisms.

## Supporting Information

Figure S1 The Organization of the ENCODE Consortium. The geographical distribution of the members of the ENCODE Consortium, with pin colors indicating the group roles as detailed in the text below.
(TIF)

Figure S2 Quantitative trait example (BCL11A). Candidates for gene regulatory features in the vicinity of SNPs at the *BCL11A* locus associated with fetal hemoglobin levels. SNPs associated with fetal hemoglobin levels are marked in red on the top line; those not associated are marked in blue. The phenotype-associated SNPs are close to an antisense transcript (AC009970.1, light orange), shown in the ENCODE gene annotations. This antisense transcript is within a region (boxed in red) with elevated levels of H3K4me1 and DNase hypersensitive sites. The phenotype-associated region is flanked by two regions (boxed in blue) with multiple strong biochemical signals associated with transcriptional regulation, including transcription factor occupancy. The data are from the lymphoblastoid cell line GM12878, as *BCL11A* is expressed in this cell line (RNA-seq track) but not in K562 (unpublished data).
(TIF)

Table S1 This supplemental table contains additional details of the computational analysis tools used by the ENCODE Consortium that are listed in Table 3. The name of each software tool appears in the first column, and subsequent columns contain the tasks for which the tool is used, the PMID reference number when available, and a web address where the tool can be accessed.
(DOC)

## Abbreviations

3C: Chromosome Conformation Capture
API: application programming interface
CAGE: Cap-Analysis of Gene Expression
ChIP: chromatin immunoprecipitation
DCC: Data Coordination Center
DHS: DNaseI hypersensitive site
ENCODE: Encyclopedia of DNA Elements
EPO: Enredo, Pecan, Ortheus approach
FDR: false discovery rate
GEO: Gene Expression Omnibus
GWAS: genome-wide association studies
IDR: Irreproducible Discovery Rate
Methyl-seq: sequencing-based methylation determination assay
NHGRI: National Human Genome Research Institute
PASRs: promoter-associated short RNAs
PET: Paired-End diTag
RACE: Rapid Amplification of cDNA Ends
RNA Pol2: RNA polymerase 2
RBP: RNA-binding protein
RRBS: Reduced Representation Bisulfite Sequencing
SRA: Sequence Read Archive
TAS: trait/disease-associated SNP
TF: transcription factor
TSS: transcription start site
