# Supplementary figures and images for: A User's Guide to the Encyclopedia of DNA Elements (ENCODE)
Source: PLoS Biol. 2011 Apr 19;9(4):e1001046. doi: 10.1371/journal.pbio.1001046 (PMC3079585; doi:10.1371/journal.pbio.1001046)

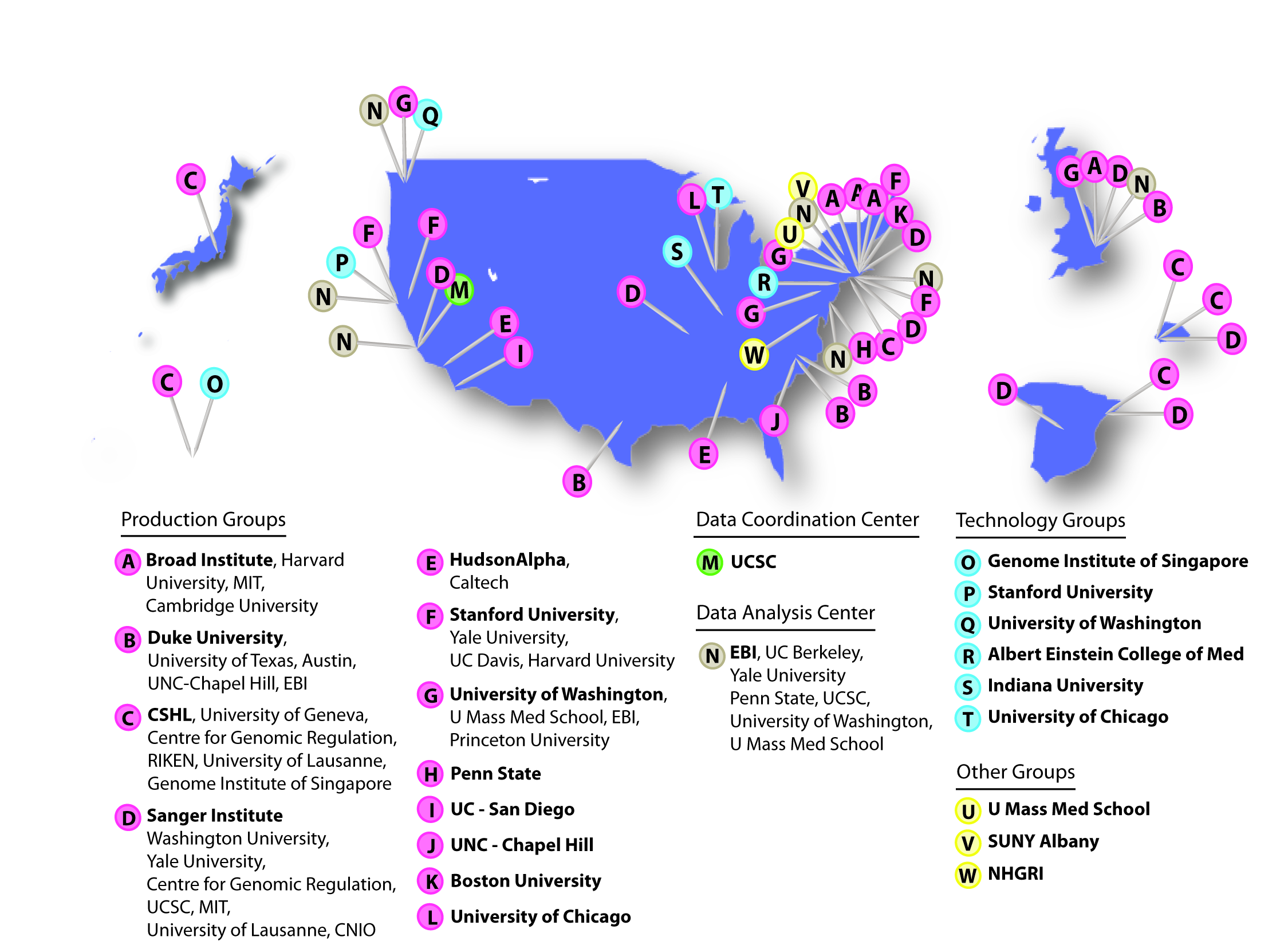

Supplement: Figure S1 — The Organization of the ENCODE Consortium. The geographical distribution of the members of the ENCODE Consortium, with pin colors indicating the group roles as detailed in the text below. (TIF) [file pbio.1001046.s001.tif]

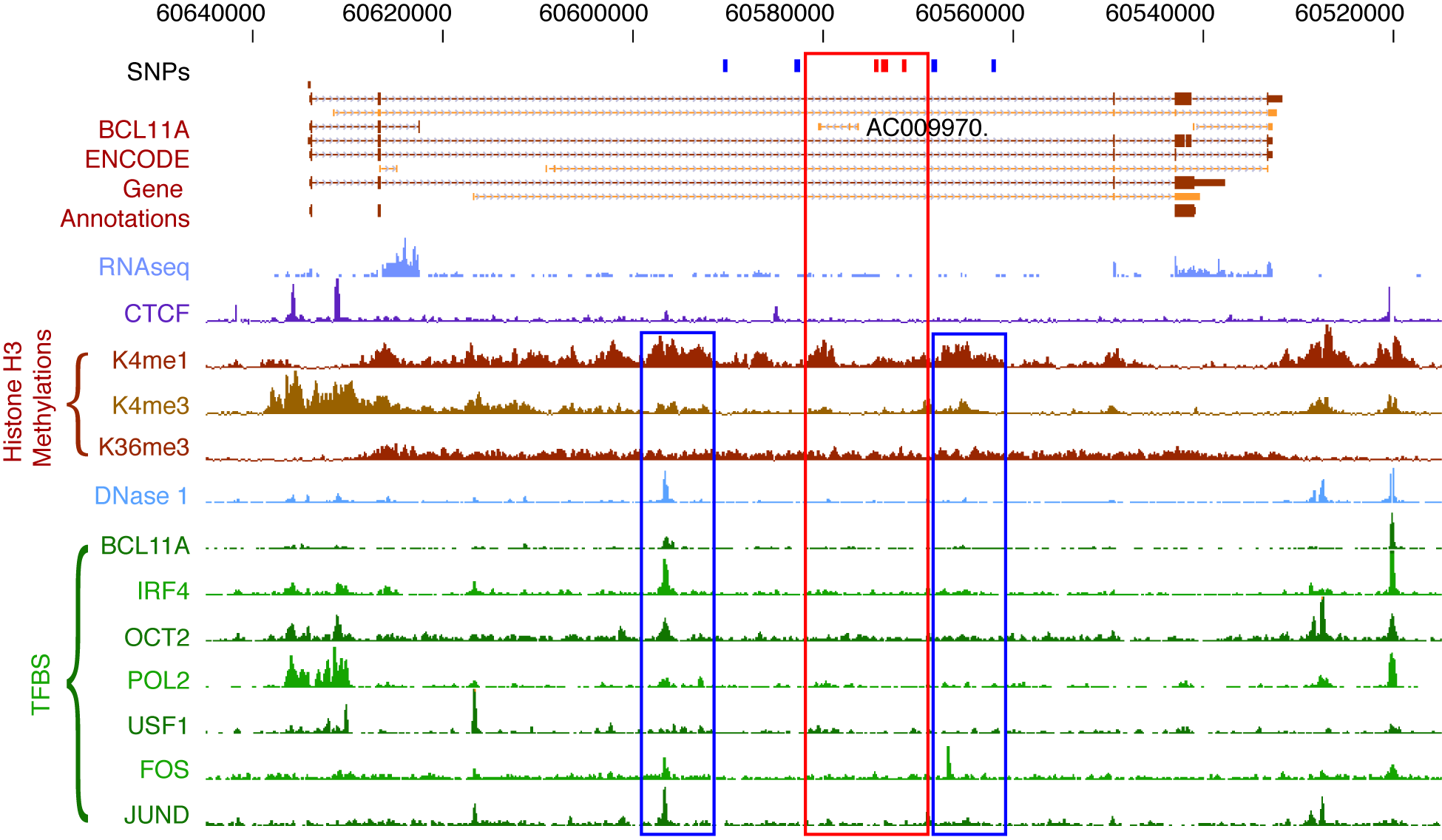

Supplement: Figure S2 — Quantitative trait example (BCL11A). Candidates for gene regulatory features in the vicinity of SNPs at the BCL11A locus associated with fetal hemoglobin levels. SNPs associated with fetal hemoglobin levels are marked in red on the top line; those not associated are marked in blue. The phenotype-associated SNPs are close to an antisense transcript (AC009970.1, light orange), shown in the ENCODE gene annotations. This antisense transcript is within a region (boxed in red) with elevated levels of H3K4me1 and DNase hypersensitive sites. The phenotype-associated region is flanked by two regions (boxed in blue) with multiple strong biochemical signals associated with transcriptional regulation, including transcription factor occupancy. The data are from the lymphoblastoid cell line GM12878, as BCL11A is expressed in this cell line (RNA-seq track) but not in K562 (unpublished data). (TIF) [file pbio.1001046.s002.tif]
