# Supplementary material for: A User's Guide to the Encyclopedia of DNA Elements (ENCODE)
Source: PLoS Biol. 2011 Apr 19;9(4):e1001046. doi: 10.1371/journal.pbio.1001046 (PMC3079585; doi:10.1371/journal.pbio.1001046)
Supplement: Table S1 — This supplemental table contains additional details of the computational analysis tools used by the ENCODE Consortium that are listed in Table 3. The name of each software tool appears in the first column, and subsequent columns contain the tasks for which the tool is used, the PMID reference number when available, and a web address where the tool can be accessed. (DOC) [file pbio.1001046.s003.doc]

**Table S1: Analysis Tools used in** the ENCODE Consortium

| **A. Short Sequence Read Alignment** | | | |
| --- | --- | --- | --- |
| **Software** | **Task** | **PMID** | **Availability** |
| Bowtie | Short read alignment | 19261174 | http://bowtie-bio.sourceforge.net/ |
| BWA | Short read alignment | 19451168 | http://bio-bwa.sourceforge.net/ |
| Delve | Pair-HMM based short read alignment | NA | Contact timolassmann@gmail.com |
| Eland | Short read alignment | NA | Part of Illumina Genome Analyzer Pipeline |
| Exonerate | Short read alignment | 15713233 | http://www.ebi.ac.uk/~guy/exonerate/ |
| GEM (GEM mapper, GEM split mapper) | Short read alignment | NA | http://gemlibrary.sourceforge.net |
| Maq | Short read alignment | NA | http://maq.sourceforge.net/ |
| Nexalign | Short read alignment | NA | http://genome.gsc.riken.jp/osc/english/software/ |
| STAR | RNA-seq alignment, *de novo* splicing. | NA | http://gingeraslab.cshl.edu/STAR/ |
| Tophat | Short read alignment and de novo splice junctions discovery | 19289445 | <http://tophat.cbcb.umd.edu/> |
|  | | | |
| **B. Signal Processing and Identification of Enriched Regions** | | | |
| **Software** | **Task** | **PMID** | **Availability** |
| align2rawsignal | Creates normalized signal from alignment | NA | http://code.google.com/p/align2rawsignal/ |
| Cufflinks | Transcript reconstruction and quantification | 20436464 | <http://cufflinks.cbcb.umd.edu/manual.html> |
| ERANGE | Peak calling for ChIP-seq and RNA-seq | 18516045 | http://woldlab.caltech.edu/rnaseq/ |
| Fluxcapacitor | Transcript quantification from RNAseq | NA | http://flux.sammeth.net/ |
| Fseq | Peak calling for DNase-seq, FAIRE-seq, and ChIP-seq | 18784119 | http://www.genome.duke.edu/labs/furey/software/fseq/ |
| Hotspot | Peak calling for DNase-seq | NA | http://www.uwencode.org/proj/hotspot-ptih |
| IQSeq | Transcript quantification from RNA-seq data | NA | <http://archive.gersteinlab.org/proj/rnaseq/IQSeq> |
| IDR | Establishing reproducible replicate thresholds | NA | http://www.encodestatistics.org/svn/idr/ |
| MACS | Peak calling for ChIP-seq | 18798982 | http://liulab.dfci.harvard.edu/MACS/ |
| MLRSeg | Detection of expressed regions from RNA-seq | NA | In preparation. |
| NextGeneId | Transcript reconstruction | NA | http://genome.crg.cat/software/geneid/index.html |
| PeakSeq | Peak calling for ChIP-seq | 19122651 | http://www.gersteinlab.org/proj/PeakSeq/ |
| QuEST | Peak calling for ChIP-seq | 19160518 | http://mendel.stanford.edu/sidowlab/downloads/quest/ |
| RSEQTools | RNASeq data processing | NA | http://rseqtools.gersteinlab.org |
| Sole-search | Peak calling for ChIP-seq | 19906703 | http://chipseq.genomecenter.ucdavis.edu/cgi-bin/chipseq.cgi |
| SPP | Peak calling for ChIP-seq | 19029915 | http://compbio.med.harvard.edu/Supplements/ChIP-seq |
| ZINBA  (Zero Inflated Negative Binomial Algorithm) | Peak calling for FAIRE | In preparation | In preparation |
|  | | | |
| **C. Integration Tools and Resources** | | | |
| **Software** | **Task** | **PMID** | **Availability** |
| ACT | Aggregate signals over genes and other features | NA | http://act.gersteinlab.org |
| AlignACE | Motif Finder | NA | http://arep.med.harvard.edu/mrnadata/mrnasoft.html |
| AnnoTrack | Track manual annotation & integrate external data | 20923551 | http://annotrack.sanger.ac.uk |
| BEDtools | BED file set operations and proximity analysis | 20110278 | <http://code.google.com/p/bedtools/> |
| BindBoost | Supervised learning framework for integrative models of TF binding | NA | Currently planned to be released in early 2011 |
| CAGT | Clustered aggregation plots over TF binding sites and other genomic features | NA | http://code.google.com/p/cagt/ |
| CHAI | Conservation analysis | 19286520 | Code available on request. |
| ChromHMM | Hidden Markov Model segmentation of functional genomics data | NA | In preparation. |
| Enredo-Pecan-Ortheus | All placental mammals whole genome alignments | 18849524 + 18849525 | http://www.ebi.ac.uk/~jherrero/downloads/enredo/  http://www.ebi.ac.uk/~bjp/pecan/  http://www.ebi.ac.uk/~bjp/ortheus/ |
| Galaxy | BED file set operations | 16169926 | http://bitbucket.org/galaxy/galaxy-central/wiki/GetGalaxy |
| Genomedata | Genome data storage and access format. | 20435580 | http://noble.gs.washington.edu/proj/genomedata/ |
| Genome Structure Correction | Robust statistical test for overlap significance. | 17571346 | http://www.encodestatistics.org/svn/genome_structural_correction/ |
| GERP v2.1 | Conservation analysis, per-base and features | 15965027 | http://mendel.stanford.edu/SidowLab/downloads/gerp/index.html |
| MEME | Motif Finder | 7584402 | http://meme.sdsc.edu/meme4_4_0/intro.html |
| MultiZ | All vertebrates whole genome alignments | 15060014 | http://www.bx.psu.edu/miller_lab/ |
| PHAST | Phylogenetic alignment. | Submitted | http://compgen.bscb.cornell.edu/phast/ |
| R | Statistical computing framework. | NA | http://www.r-project.org/ |
| Segtools | Plotting and Tabulating segmentation data | NA | http://noble.gs.washington.edu/proj/segtools/ |
| Segway | Dynamic Bayesian network segmentation of multitrack  functional genomics data | In preparation | http://noble.gs.washington.edu/proj/segway/ |
| Weeder | Motif Finder | 15215380 | http://159.149.109.9/modtools/ |
